# Rehabilitation for spinal muscular atrophy patients in China: a national cross-sectional study

**DOI:** 10.1186/s13023-024-03291-x

**Published:** 2024-07-25

**Authors:** Duan Wang, Ting Zhang, Yi Li, Jiayu Liu, Yongzhu Jia, Nong Xiao

**Affiliations:** Department of Rehabilitation, Children’s Hospital of Chongqing Medical University, National Clinical Research Center for Child Health and Disorders, Ministry of Education Key Laboratory of Child Development and Disorders, Chongqing Key Laboratory of Child Neurodevelopment and Cognitive Disorders, Chongqing, 400014 China

**Keywords:** Spinal muscular atrophy, Rehabilitation, Cross-sectional study, PedsQL

## Abstract

**Background:**

The management of Spinal Muscular Atrophy (SMA) requires a multidisciplinary treatment approach, wherein rehabilitation constitutes an integral element. In this study, we examined the effects of rehabilitation among Chinese SMA patients and assessed the real-world efficacy of rehabilitation interventions.

**Methods:**

We conducted a cross-sectional online survey on SMA patients from June 9, 2023, to June 30, 2023, through the Meier Advocacy & Support Center using data from the Center’s database and electronic questionnaires. The rehabilitation situation of the participants over the past 14 months were investigated. Logistic binary regression was used to analyze the relationship between Pediatric Quality of Life Inventory(PedsQL™) scores and rehabilitation.

**Result:**

A total of 186 questionnaires were finally analyzed. Only 29 patients did not rehabilitated in the past 14 months. A significant correlation between age and type of rehabilitation, as well as between age and duration of rehabilitation. Patients receiving no rehabilitation or solely home-based rehabilitation exhibited a higher median age of 8.4 compared to those undergoing standard rehabilitation or a combination of standard and home-based rehabilitation, with a median age of 4.9 (z-score = -4.49, *p*-value < 0.001). In addition, long-term rehabilitation (OR = 0.314, 95%CI = 0.106–0.927, *p* = 0.04) were negatively correlated with lower PedsQL™ Neuromuscular Module scores, and PedsQL scores in the long-term rehabilitation group were higher than those in the short-term and no-rehabilitation groups (54.2 ± 15.1 vs. 45.9 ± 14.4 and 42.3 ± 14.3, *p* = 0.01), with the most significant difference observed in the physical function section (59.0 ± 15.8 vs. 46.8 ± 15.2 and 45.6 ± 15.9, *p* < 0.01). Mobility and exercise (OR = 0.26, 95%CI = 0.08–0.81, *p* = 0.02), as well as assistive technology (OR = 0.28, 95%CI = 0.10–0.82, *p* = 0.02), were independently associated with a lower score in a negative direction.

**Conclusion:**

The study found that long-term rehabilitation was linked to higher PedsQL scores in SMA patients, highlighting the need for standardized rehabilitation programs to enhance function and quality of life.

**Supplementary Information:**

The online version contains supplementary material available at 10.1186/s13023-024-03291-x.

## Background

Spinal muscular atrophy (SMA) is a rare neuromuscular disease characterized by autosomal recessive inheritance and caused by biallelic loss of the survival motor neuron 1 (*SMN1*) gene, which primarily affects motor neurons in the anterior medullar horn and brainstem, leading to their apoptosis [1]. SMA manifests across a spectrum of phenotypes, ranging from severely compromised neonates and infants to adults with minimal symptoms. The classification of SMA types is typically based on age at onset and the attainment of specific motor milestones, resulting in at least four distinct categories [2]. SMA type 1 is the most severe form, leading to infant mortality without respiratory support. Symptoms include muscle weakness, feeble crying, breathing and feeding issues. SMA type 2 is moderately severe, with individuals unable to stand or walk. SMA type 3 is milder, allowing independent walking [3].

Currently, the development of modifying drugs such as Nusinersen, Risdiplam, and Onasemnogene abeparvovec has significantly improved the life expectancy and motor function of patients with SMA [4–7]. However, it is important to fully optimize the existing functional capacities to enhance the overall quality of life for these patients.

Clinicians place a significant emphasis on a multi-disciplinary treatment (MDT) approach for the management of SMA patients, with rehabilitation serving as an integral component. Rehabilitation interventions encompass a range of techniques such as stretching, positioning, mobility exercises, and chest physiotherapy, etc., tailored to individual patient needs to enhance functionality and alleviate symptoms [8–10]. Numerous studies have demonstrated that rehabilitation training, encompassing strength exercises [11, 12], physical therapy [13] and comprehensive training [14], can lead to improvements in motor function. Additionally, physical modalities such as electrical stimulation [15] and the utilization of assistive technology (AT) [16, 17] also play a pivotal role in enhancing the quality of life for SMA patients. However, there has been limited research conducted on the rehabilitation of Chinese SMA patients and the relationship between rehabilitation treatment and their quality of life. The objective of this study is to examine the prevailing rehabilitation strategies utilized by individuals with SMA in China and assess the practical impact of rehabilitation on improving their overall quality of life.

## Materials and methods

### Study design and population

A cross-sectional online survey was conducted between June 9, 2023, and June 30, 2023, at the Meier Advocacy & Support Center, which is the first civil affairs registered non-profit organization in mainland China that focuses on the treatment and management of SMA. The survey was conducted using ‘Questionnaire Star,’ a platform designed for creating, distributing, and retrieving mobile questionnaires. Briefly, the questionnaire was distributed to parents of SMA patients who were members of the Meier Advocacy & Support Center’s WeChat group.

The inclusion criteria consisted of individuals with a confirmed diagnosis of SMA as determined by genetic analysis, who provided informed consent for voluntary participation. Exclusion criteria encompassed individuals over the age of 18 years and questionnaires deemed to be invalid.

This study was approved by the Ethics Committee of the Children’s Hospital of Chongqing Medical University (approval No. 2023 − 404) and informed consent was obtained from all patients’ parents or legal guardians.

### Data collection

Data were collected from two primary sources. Firstly, patient information, including age, gender, SMA type, onset age and residence location, was obtained by matching patients with records in the Meier Advocacy & Support Center database. The second part of the data was gathered through an electronic questionnaire (Supplementary Material 1), designed by a collaborative team consisting of senior doctors from the rehabilitation, pediatrics and neurology departments, and rehabilitation therapists at the Children’s Hospital of Chongqing Medical University and Meier Advocacy & Support Center. The questionnaire encompassed three sections: (1) basic information, covering medication, family income, parental education, daily caregiving duration, and patient functionality; (2) rehabilitation details, including duration, location, specific activities and the challenges encountered; and (3) patient-reported outcomes, using the Pediatric Quality of Life Inventory: PedsQL™ Infant Scales for patients aged 1–24 months and PedsQL™ Neuromuscular Module (NMM) for patients aged 2–18 years. The PedsQL NMM has demonstrated responsiveness to impaired function in previous studies [18–21], and the PedsQL Infant Scales have exhibited good reliability and construct validity across diverse populations for patients aged 1–24 months [22].

Quality control measures involved excluding invalid questionnaires, which included those with repetitive or incomplete responses and completion times less than 4 min.

### Group definition

In this study, we classified rehabilitation conducted in hospitals or institutions as “standard rehabilitation,” while rehabilitation performed at home was categorized as “family rehabilitation”. To assess the rehabilitation history of patients over the past 14 months before the survey, we classified them into three distinct groups: the long-term rehabilitation group (LR group), the short-term rehabilitation group (SR group), and the no rehabilitation group (NR group). The LR group comprised patients who had engaged in standard rehabilitation for a minimum of 7 months (involving training at least once a week for a consecutive 4-week period within the month) or had a home rehabilitation frequency of at least 3 days per week. Patients who had never undergone any form of rehabilitation were placed in the NR group, while the remaining patients were categorized as part of the SR group.

### Statistical analysis

Statistical analysis was performed using the SPSS 25.0 software package (SPSS for Windows, IBM Corp., Armonk, NY, USA). The normality of the data was assessed using the Kolmogorov-Smirnov test. Measurement data are presented as either mean ± standard deviation (SD) or median (interquartile range), while count data are expressed as frequencies or percentages. To evaluate data correlations or differences, the chi-square test and single-sample t-test/ANOVA were performed, with logistic binary regression used to analyze relevant factors. A significance level of *p* < 0.05 (two-sided) was considered statistically significant.

## Results

**Fig. 1:**
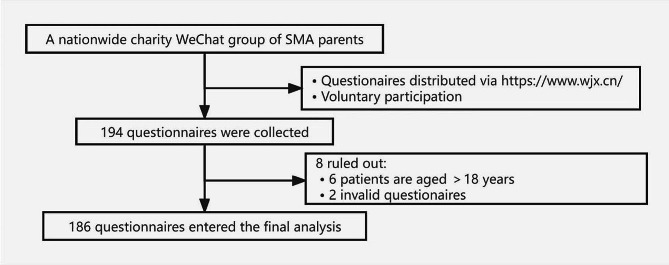
Flow chart showing the distribution and collection of questionnaires

This study included a total of 194 participants diagnosed with SMA. However, considering that six participants were aged over 18 years and two questionnaires were determined to be invalid, a total of 186 questionnaires were used for final analysis (Fig. 1). Participants were recruited on a national scale (Supplementary Material 2). The average age of the participants was 6.70 ± 3.81 years, with males comprising 51.6% of the cohort. Regarding SMA types, 12.9% were classified as type 1, 71.0% as type 2, and 16.1% as type 3.

### The demographics and rehabilitation situation of SMA patients

**Fig. 2:**
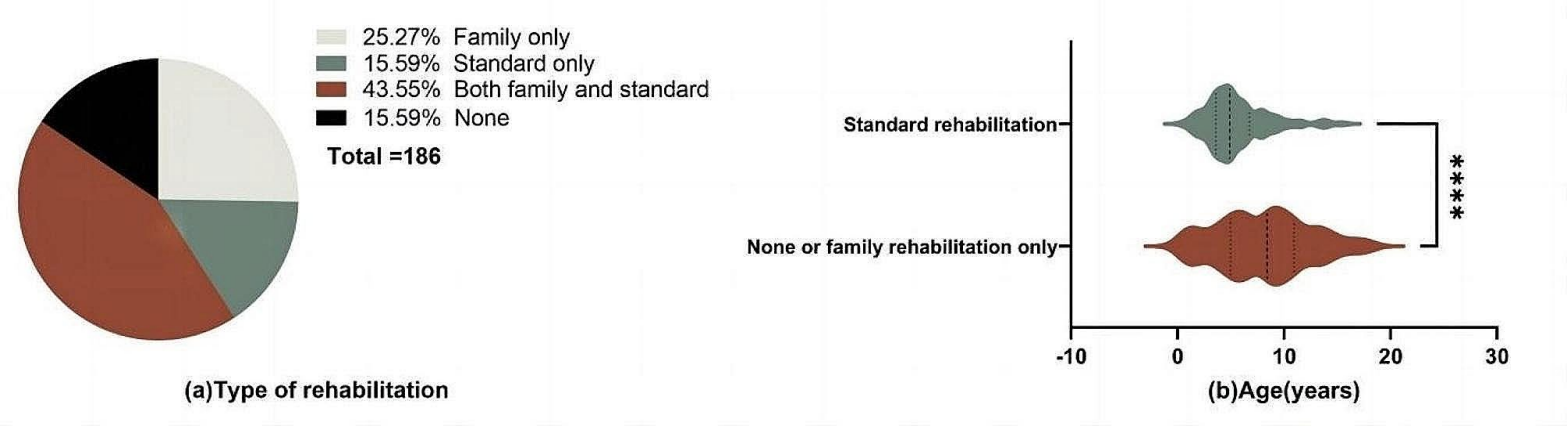
Rehabilitative types of SMA

**Fig. 3:**
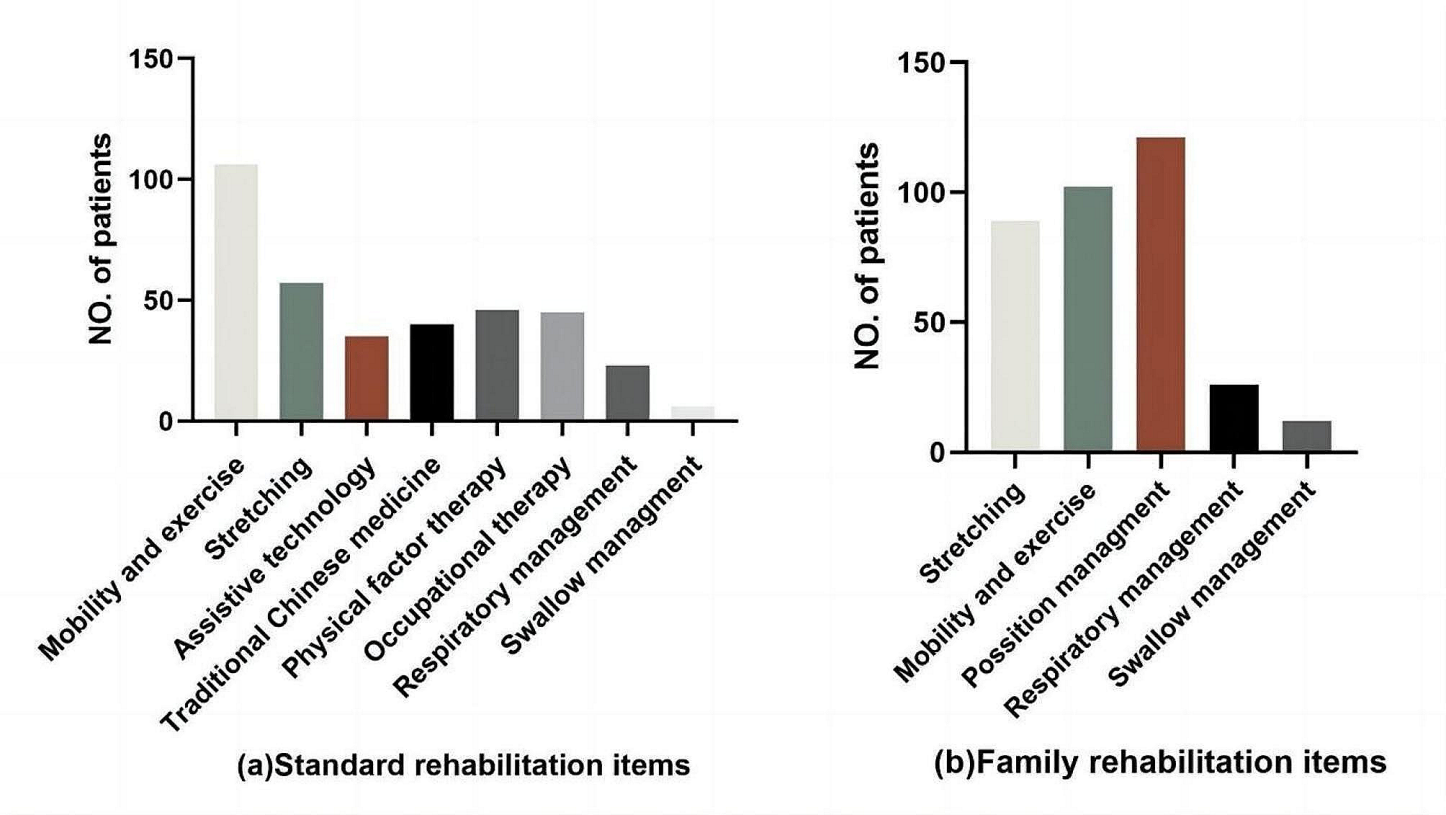
Rehabilitative items of SMA patients

As shown in Fig. 2(a), of the 186 patients, 43.55% (*n* = 81) underwent rehabilitation both at home and in a hospital/institution, 25.27% (*n* = 47) received rehabilitation at home only and 15.59% (*n* = 29) opted for standard training alone, while 29 did not undergo any rehabilitation in the past 14 months. Among these 29 patients, the primary reason for not pursuing rehabilitation were economic constraints (66.2%) and a lack of family rehabilitation skills (47.6%). The mean age of patients varied depending on the type of rehabilitation they received, and the mean ages for those undergoing family and standard rehabilitation, standard rehabilitation alone, family-based rehabilitation alone, and no rehabilitation were 5.40 ± 2.82, 5.90 ± 3.01, 7.48 ± 4.43, and 9.31 ± 3.90, respectively. As illustrated in Fig. 2(b), there was a significant age difference between those who received no rehabilitation or family-based rehabilitation only (in red, median = 8.4 years) and those who received standard rehabilitation or both family and standard rehabilitation (in green, median = 4.9 years) (z=-4.49, *p* < 0.001). Figure 3 provides an overview of the specific components of standard and family rehabilitation. The most frequently addressed rehabilitation elements for SMA patients included mobility and exercise, stretching, position management, and the use of assistive technology.

In this study, a total of 145 patients were stratified into three groups: the LR group (*n* = 104), SR group (*n* = 34), and NR group (*n* = 7). Comparative analysis of the demographics and clinical characteristics among these groups is shown in Table 1. The results indicate that the proportion of patients undergoing gene therapy was significantly lower in the NR group compared to the LR and SR groups (71.4% vs. 96.6% and 94.1%, *p* = 0.01). Furthermore, patients in the SR group (mean age = 8.43 ± 3.98) and NR group (mean age = 9.51 ± 4.44) were found to be older compared to those in the LR group (mean age = 6.16 ± 3.58). We observed that this age discrepancy corresponded with the distribution of SMA types, as a higher percentage of type III patients were observed in the SR group in contrast to the LR group (41.2% vs. 9.7%). A similar trend was observed in patients who could walk independently (12.4% vs. 35.3%, *p*<0.01). Additionally, it was observed that patients requiring respiratory ventilation were more inclined to short-term rehabilitation (2.8% vs. 14.7%, *p* = 0.01). Nevertheless, no significant differences were observed among the LR, SR and NR groups concerning gender, bone or joint deformity, nasogastric tube intubation, annual family income, parental educational background, and the duration of daily caregiving.

**Table 1:** Demographics and characteristics of SMA patients with different rehabilitated duration

Variables	LR group (*n* = 145)	SR group (*n* = 34)	NR group (*n* = 7)	*p*-value
**Age (years**,** mean ± SD)**	6.16 ± 3.58	8.43 ± 3.98	9.51 ± 4.44	<0.01
**Male (%)**	79(54.5%)	14(41.2%)	3(54.5%)	0.34
**Rehabilitation age (years**,** mean ± SD)**	2.98 ± 2.60	3.73 ± 2.24	-	0.34
**Gene therapy (%)**	140(96.6%)	32(94.1%)	5(71.4%)	0.01
**SMA type (%)**				< 0.01
I type	17(11.7%)	5(14.7%)	2(28.6%)	
II type	114(78.6%)	15(44.1%)	3(42.9%)	
III type	14(9.7%)	14(41.2%)	2(28.6%)	
**Best motor function (%)**				< 0.01
Unable sit	19(13.1%)	5(14.7%)	3(42.9%)	
Sit	108(74.5%)	17(50.0%)	2(28.6%)	
Walk	18(12.4%)	12(35.3%)	2(28.6%)	
**Bone or joint deformity(%)**	126(86.9%)	27(79.4%)	5(71.4%)	0.32
**Nasogastric tube intubation(%)**	3(2.1%)	1(2.9%)	0	0.88
**Ventilator support(%)**	4(2.8%)	5(14.7%)	0	0.01
**Annual family income (thousands**,** %)**				0.94
< 50	66(45.5%)	16(47.1%)	4(57.1%)	
50–100	51(35.2%)	13(38.2%)	2(28.6%)	
> 100	28(19.3%)	5(14.7%)	1(14.3%)	
**Education background of parents(%)**				0.19
Junior school or below	38(26.2%)	10(29.4%)	3(42.9%)	
High school	23(15.9%)	10(29.4%)	2(28.6%)	
College or higher	84(57.9%)	14(41.2%)	2(28.6%)	
**Duration of care per day(%)**				0.87
Full-time	116(80.0%)	28(82.4%)	6(85.7%)	
Half-time	15(10.3%)	5(11.8%)	1(14.3%)	
Short-time or NA	14(9.7%)	2(4.9%)	0	

LR group: Long-term rehabilitation group; SR group: Short-term rehabilitation group; NR group: No rehabilitation group

### The PedsQL score of SMA patients

Among the 186 patients included in the study, the mean PedsQL MNN score for patients aged above 2 years (*n* = 170) was 55.5 ± 1.5, whereas the PedsQL infant scale (*n* = 16) yielded a score of 71.3 ± 8.8. Notably, all patients aged below 2 years had received gene therapy, while only 9 patients above 2 years had not undergone gene therapy. The study observed that patients who received gene therapy had significantly higher PedsQL MNN scores compared to those who did not receive it (41.56 ± 13.48 vs. 56.27 ± 15.70, *p* = 0.007). Consequently, further analysis was conducted specifically among the subset of patients who received gene therapy (*n* = 161). As presented in Table 2, the analysis revealed strong associations between the PedsQL MNN score and factors such as age, SMA type, best motor function, bone or joint deformity, and long-term rehabilitation. However, no significant differences were found in gender, nasogastric tube intubation, ventilator support, and annual family income between the two groups. Subsequently, Table 3 outlines the results of binary logistic regression about factors independently correlated with a low PedsQL MNN score. The analysis indicated that walking ability was negatively correlated with a lower PedsQL MNN score (OR = 0.027, 95%CI = 0.002–0.375, *p* < 0.01). Similarly, a negative correlation was observed in the long-term rehabilitation group (OR = 0.314, 95%CI = 0.106–0.927, *p* = 0.04). Conversely, bone or joint deformity was positively associated with a lower PedsQL MNN score (OR = 3.786, 95%CI = 1.156–12.397, *p* = 0.03).

**Table 2:** Factors associated with PedsQL MNN score in patients with gene therapy(*N* = 161)

Variables	PedsQL MNN < 60 (*n* = 89)	PedsQL MNN ≥ 60 (*n* = 72)	*p*-value
**Age (years**,** mean ± SD)**	7.49 ± 3.72	6.54 ± 3.24	0.09
**Male (%)**	44(49.9%)	41(56.9%)	0.34
**SMA type (%)**			0.02
I type	11(12.4%)	1(1.4%)	
II type	64(71.9%)	55(76.4%)	
III type	14(15.7%)	16(22.2%)	
**Best motor function (%)**			< 0.01
Unable sit	13(14.6%)	1(1.4%)	
Sit	66(74.2%)	49(68.1%)	
Walk	10(11.2%)	22(30.6%)	
**Bone or joint deformity (%)**	83(93.3%)	57(79.2%)	< 0.01
**Nasogastric tube intubation (%)**	2(2.2%)	1(1.4%)	0.69
**Ventilator support(%)**	5(5.6%)	2(2.8%)	0.38
**Annual family income (thousands**,** %)**			0.28
< 50	44(49.4%)	27(37.5%)	
50–100	33(37.1%)	31(43.1%)	
> 100	12(13.5%)	14(19.4%)	
**Long-term rehabilitation**	65(73.0%)	61(84.7%)	0.07

**Table 3:** Factors independently associated with a low PedQL MNN score (< 60) in patients with gene therapy (*N* = 161)

Variables	OR (95%CI)	*p*-value
**Age**,** years**	1.012(0.898–1.141)	0.84
**SMA type (%)**		
I type	Ref	
II type	0.185(0.019–1.824)	0.15
III type	0.493(0.031–7.726)	0.61
**Best motor function (%)**		
Unable sit	Ref	
Sit	0.174(0.019–1.585)	0.12
Walk	0.027(0.002–0.375)	<0.01
**Bone or joint deformity (%)**	3.786(1.156–12.397)	0.03
**Long-term rehabilitation**	0.314(0.106–0.927)	0.04

### Relevance of rehabilitation and PedsQL MNN in unable-to-walk patients with gene therapy

**Fig. 4:**
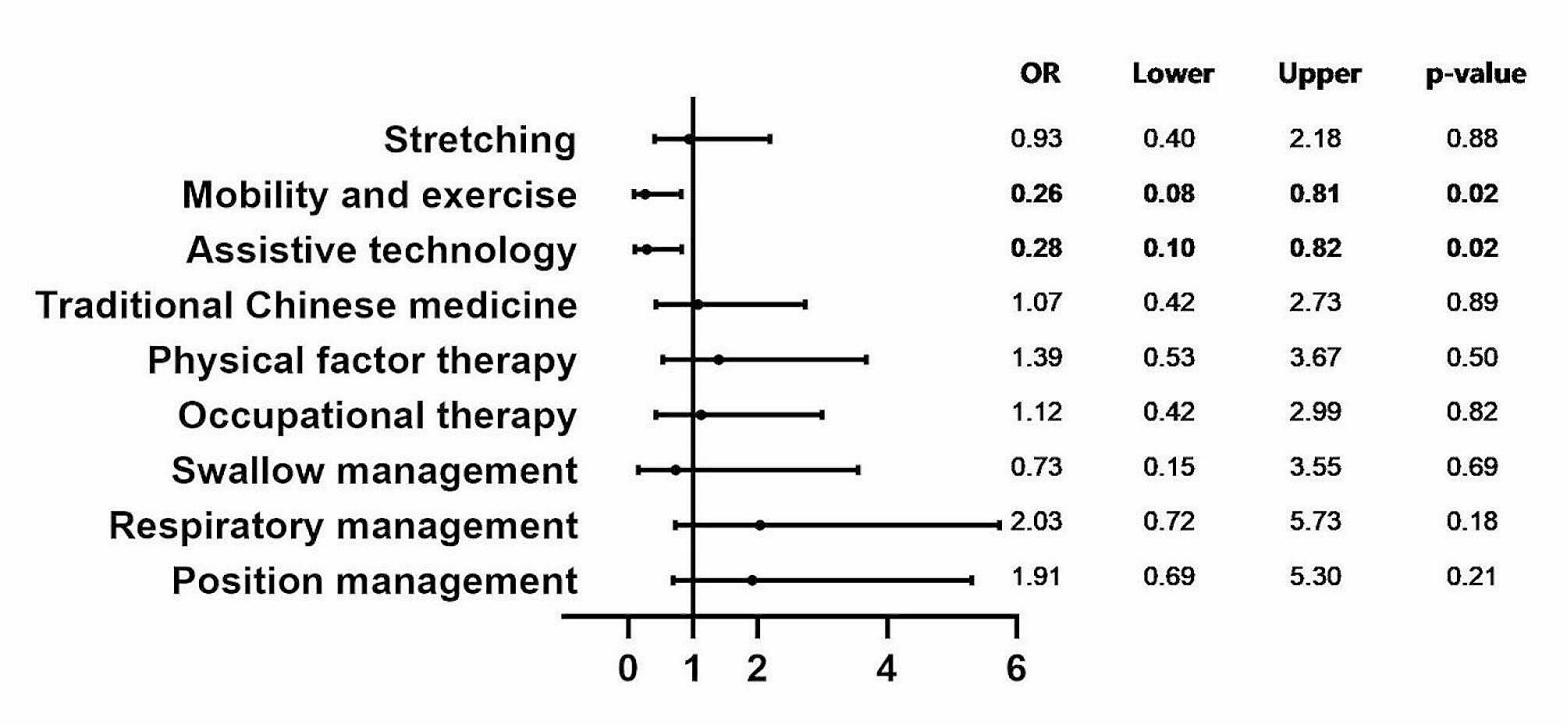
Forest plot of physical function score < 60 patients and rehabilitation items (in unable-to-walk patients with gene therapy, *N* = 129)

Given the strong correlation between walking ability and the PedsQL MNN score, we conducted subgroup analysis using data from patients who were unable to walk. As shown in Table 4, the LR group exhibited a higher total score compared to the SR and NR groups (54.2 ± 15.1 vs. 45.9 ± 14.4 and 42.3 ± 14.3, *p* = 0.01), which was particularly prominent in the physical function section (59.0 ± 15.8 vs. 46.8 ± 15.2 and 45.6 ± 15.9, *p* < 0.01). Conversely, no significant differences were observed among the three groups concerning communication and family resources. Furthermore, we assessed the relationship between rehabilitative items and the physical function score (Fig. 4), and the results indicated that mobility and exercise (OR = 0.26, 95%CI = 0.08–0.81, *p* = 0.02), as well as assistive technology (OR = 0.28, 95%CI = 0.10–0.82, *p* = 0.02), were independently associated with a lower score in a negative direction, while no significant differences were observed in the other items.

**Table 4:** Impact of rehabilitation treatment on PedsQL scores in unable-to-walk patients with gene therapy (*N* = 129)

Variables	LR group (*n* = 108)	SR group (*n* = 18)	NR group (*n* = 3)	*F*	*p*
**Physical function (mean ± SD)**	59.0 ± 15.8	46.8 ± 15.2	45.6 ± 15.9	5.42	<0.01
**Communication (mean ± SD)**	74.6 ± 26.4	64.4 ± 29.7	55.6 ± 29.3	1.74	0.18
**Family resources (mean ± SD)**	32.5 ± 22.4	31.7 ± 24.0	23.3 ± 15.3	0.25	0.78
**Total score (mean ± SD)**	54.2 ± 15.1	45.9 ± 14.4	42.3 ± 14.3	4.58	0.01

LR group: Long-term rehabilitation group; SR group: Short-term rehabilitation group; NR group: No rehabilitation group

## Discussion

In this study, we reviewed the rehabilitation progress of 186 SMA patients for 14 months. Patient age influenced the type and duration of rehabilitation. Among gene therapy recipients, those in long-term rehabilitation had higher PedsQL MNN scores compared to those in short-term or no rehabilitation. Exercise and AT were found to improve scores, especially in patients unable to walk. To our best knowledge, this study is the first to examine the impact of rehabilitation on PedsQL scores in SMA patients in China.

Our study revealed that only 15.59% of SMA patients did not engage in rehabilitation, a rate lower than that reported in other domestic studies [3], which could potentially be attributed to differences in the study population, as parents associated with SMA Care Centers may exhibit increased concerns regarding their children’s management. Our findings also highlighted a significant correlation between age and type, as well as age and duration of rehabilitation. Importantly, it is worth considering that prolonged hospital-based rehabilitation may not always be optimal, especially for school-age children. SMA patients, on the whole, tend to maintain their general intelligence and language comprehension [23], enabling many of them to continue their education and social activities [24]. As patients age, there tends to be a shift from hospital-centered to home-based rehabilitation, as well as a transition from long-term to short-term rehabilitation, which allows patients to allocate more time to their school and social lives, aligning with our current findings. Furthermore, rehabilitation plans are individualized to cater to the specific needs and functional abilities of each patient, and those with better functional preservation, such as those with SMA type III who can walk, may require less frequent and shorter-duration rehabilitation sessions.

The findings of our present study revealed that the mean PedsQL MNN scores were 55.5 ± 1.5, consistent with previously reported results in the literature [25, 26]. Our study has shown that patients who underwent gene therapy exhibited notably elevated PedsQL scores in comparison to those who did not receive this treatment, providing additional evidence of the efficacy of gene therapy. The established effectiveness [20, 27] of gene therapy in extending patients’ lifespans and improving motor function has significantly impacted the progression of SMA [28–30]. These findings indicate that the initial classification of SMA types may not consistently predict the ultimate exercise outcome, as the latter is more closely linked to the patient’s overall survival prognosis. Our study further revealed a significant correlation between patients’ quality-of-life assessments and their maximal motor function, rather than their specific SMA subtype. The capacity to walk emerged as a crucial determinant in sustaining a favorable quality of life among individuals with SMA. Moreover, the prevalence of bone and joint deformities, commonly seen in individuals with SMA, particularly affecting the ankle, knee, and spine [3], was recognized as a significant factor affecting PedsQL. These deformities restrict joint range of motion and lead to decreased mobility. Despite progress in early intervention with disease-modifying treatments, addressing joint contractures remains a difficult task in certain patients with advanced SMA [31]. Numerous studies have confirmed the positive impact of rehabilitation training on SMA patients [13, 14]. However, limited research has been done on the duration of training. Our study found that patients in the LR group achieved significantly higher PedsQL scores than SR group and NR group, particularly in terms of physical function. In line with the SMA rehabilitation consensus [8], it is recommended to undergo physical therapy sessions at a frequency exceeding 5 times per week to enhance the quality of life. Therefore, adherence to an adequate frequency of rehabilitation is important for improving the quality of life in SMA patients. Several animal experiments have demonstrated the long-term benefits of exercise in SMA-like mice [32–35]. Additionally, a progressive resistance training program was found to be feasible, safe, and well-tolerated in children with SMA [11]. The utilization of AT, such as traditional wheelchairs, customized seats and standing frames, directly contributes to improving the quality of life by enabling autonomous movement and hand operation for patients, and the use of some robotic exoskeletons to improve hip, knee and ankle ROM and maximum isometric strength has been reported to be well tolerated [16, 17, 36]. Consistent with those findings, our results shown that exercises and AT may play a pivotal role in enhancing motor function and overall life portability. Acupuncture has been reported in previous literature to have a synergistic effect in sports and exercise and can help improve patients’ spasticity [37]. However, in our study, traditional Chinese medicine treatments did not significantly improve patients’ quality of life independently. Other studies suggest that certain physical factor treatments, such as electrical stimulation, can be beneficial in enhancing motor function [15]. Nevertheless, in our study, no significant differences in quality of life were observed between physical factor treatments such as magnetic stimulation and electrical stimulation, which could be attributed to differences in treatment parameters across various centers.

Limitations of the study: firstly, the survey was conducted at the Meyer SMA Care Center, where most of the registered individuals had a higher level of education or placed a greater emphasis on their children’s health management, and thus, it could be possible that these data may not fully represent severely ill patients or those with SMA who are not receiving adequate attention from their parents. However, despite this potential limitation, the findings still provide valuable insights into the relationship between rehabilitation and PedsQL. Secondly, the present study lacked data on the number of *SMN2* copies, which could potentially impact the functional prognosis of patients, thereby providing further insights into the heterogeneity of the patient population.

## Conclusion

In China, as SMA patients age, there was a growing trend towards family rehabilitation with shorter durations. Long-term rehabilitation has been associated with improved quality of life, although further research is necessary to establish causation. Early initiation of standardized rehabilitation is essential for providing optimal care.

### Electronic supplementary material

Below is the link to the electronic supplementary material.

Supplementary Material 1: Questionnaire

Supplementary Material 2: The national distribution of patients

## Data Availability

The datasets used and analyzed during the current study are not openly available due to privacy concerns. However, interested individuals can request access to the datasets from the corresponding author, and their reasonable requests will be considered.

## Abbreviations

SMA: Spinal muscular atrophy
SMN1: Survival motor neuron 1
MDT: Multi-disciplinary treatment
AT: Assistive technology
PedsQL™ NMM: PedsQL™ Neuromuscular Module
LR group: Long-term rehabilitation group
SR group: Short-term rehabilitation group
NR group: No rehabilitation group
SD: Standard deviation
