# Supplementary material for: Rehabilitation for spinal muscular atrophy patients in China: a national cross-sectional study
Source: Orphanet J Rare Dis. 2024 Jul 25;19:279. doi: 10.1186/s13023-024-03291-x (PMC11282710; doi:10.1186/s13023-024-03291-x)
Supplement: Supplementary file 1 — Supplementary Material 1: Questionnaire [file 13023_2024_3291_MOESM1_ESM.docx]

Supply material 1

**Questionnaire on the rehabilitation treatment of spinal muscular atrophy (SMA) patients**

**Introduction:** Dear friends, this is a questionnaire about the rehabilitation treatment of SMA patients, aiming to look into the rehabilitation status of SMA patients in China, and to explore the relationship between rehabilitation and functional outcomes. Thank you for participating in this survey and make a contribution to the "confrontation" SMA!

**Statement:** This questionnaire targets to 0-18 years old SMA patients, which is reported by the parents. It included three parts: basic information, rehabilitation situation and the patient report outcome. It will take about 5-8 minutes to complete the questionnaire. Please answer according to the actual situation of the child.

**Privacy Statement:** This questionnaire is only used for research. All your information will be strictly confidential. We mark individuals with serial number, evaluate the whole population and do not involve individual sensitive information.

**Participation in this study is your choice and you can decide to quit at any time**

**Part 1: Basic information**

**1. Your QQ number registered in Meier: [fill in the blank question]***__________________________

**2. Have the patient received Nusinersen treatment[single choice] ***

| ○ Yes, please input the number of intrathecal injection therapy: __________times * |
| --- |
| ○ No |

**3. Have the patient received risdiplam treatment[single choice] ***

| ○ Yes, the data of first administration is: _______ * |
| --- |
| ○ No |

**4. Have the patient participated in the clinical trial project of injection gene therapy (e. g. EXG001-307, GC101, OAV-101) [single choice] ***

| ○ Yes, please input the number of therapy: __________times * |
| --- |
| ○ No |

**5. Your annual** **family income [single choice] ***

| ○ <50,000 yuan |
| --- |
| ○ 50,000-100,000 yuan |
| ○ 100,000-200,000 yuan |
| ○ > 200,000 yuan |

**6. Your education background [Single choice] ***

| ○ Junior high school or below |
| --- |
| ○ High School |
| ○ University undergraduate / junior college |
| ○ Graduate student or above |

**7. The duration of daily care for patient per day is [single choice] ***

| ○ Full-time care (> 16 hours / day) |
| --- |
| ○ Half-time care (8-16 hours / day) |
| ○ Short-time care (<8 hours / day) |
| ○ does not need to be looked after |

**8. The best motor milestone for the patient [single choice] ***

| ○ Can't sit |
| --- |
| ○ Sit alone (without hands or arms to balance and sit for at least 10 seconds) |
| ○ Walk alone (at least 10 meters without assistance) |

**9. Accompanied by bone/joint deformity or contracture (e. g., scoliosis, or any joint contracture, deformation of hip, knee, ankle, toe, shoulder, elbow, wrist and so on) [Single choice] ***

| ○ yes |
| --- |
| ○ No |

**10 whether a feeding tube was placed or a gastrostomy [single choice] ***

| ○ yes |
| --- |
| ○ No |

**11. Use ventilator-assisted ventilation (including invasive and non-invasive)? [Single choice] ***

| ○ Almost full time (> 20 hours / day) |
| --- |
| ○ Most of the time (16-20 hours / day) |
| ○ only when sleeping |
| ○ No |

**Part 2: Rehabilitation situation**

**1. Do you and the patient have the intention of rehabilitation treatment?[single choice]***

| ○ Yes, we have |
| --- |
| ○ No, we have not |

**2. Whether the patient has received** **standard rehabilitation treatment (including the hospital rehabilitation center or private rehabilitation institutions)?[single choice]***

| ○ yes |
| --- |
| ○ No |

**3. The first time of standard rehabilitation is: ___ year ___ month** **[fill in the blank question] ***

Depends on option 1 in question 2

1. **How many months have the patient received rehabilitation from a** **standard institution in the past 14 months(May 2022 to now June 2023)?**

**(Tip:** **As long as there is one day of rehabilitation within the month). [single choice] ***

| ○ 1 month |
| --- |
| ○ 2 months |
| ○ 3 months |
| ○ 4 months |
| ○ 5 months |
| ○ 6 months |
| ○ 7 months |
| ○ 8 months |
| ○ 9 months |
| ○ 10 months |
| ○ 11 months |
| ○ 12 months |
| ○ 13 months |
| ○ 14 months |

Depends on option 1 in question 2

1. **In a single training month, how many weeks per month the child rehabilitated in a professional institution?**

**(Tip: As long as there is one day of rehabilitation within the week, and select the most common situation) [Single choice] ***

| ○ 1 week |
| --- |
| ○ 2 weeks |
| ○ 3 weeks |
| ○ 4 weeks |

Depends on option 1 in question 2

**6. In a single** **training week, how many days per week the child rehabilitated in professional institution: (Tip: Select the most common situation)[Single choice] ***

| ○ <3 days |
| --- |
| ○ 3-5 days |
| ○> 5 days |

Depends on option 1 in question 2

**7. The locations of the patient rehabilitate are mostly in[multiple choice] ***

| □ Rehabilitation center of a tertiary hospital |
| --- |
| □ Rehabilitation center of the secondary hospital |
| □ Private rehabilitation facility |

Depends on option 1 in question 2

**8.** **What rehabilitation treatment has your child conducted in the rehabilitation center?[multiple choice]***

| □ Physical factor therapy (such as electrical stimulation, magnetic stimulation, etc.) |
| --- |
| □ Stretch training |
| □ Motor training |
| □ Respiratory function training (autonomous cough training, auxiliary sputum discharge training, respiratory muscle training, etc.) |
| □ Swallowing function training / oral sensory stimulation |
| □ Use auxiliary equipment or wear orthotics |
| □ Occupational therapy |
| □ Psychotherapy |
| □ nutritional care |
| □ Traditional Chinese medicine therapy (acupuncture etc.) |
| □ Other _________________* |

Depends on option 1 in question 2

**9. The types of assistive device your child using now include [multiple choice] ***

| □ Pressure sore cushion (molding pillow, wedge pad etc.) |
| --- |
| □ Posture correcting chair |
| □ standing frame |
| □ Chest / neck brace |
| □ Ankle foot orthoses (including corrective shoes, ankle foot orthoses, knee and ankle foot orthoses, etc.) |
| □ Additional trunk support parts to other wheelchairs |
| □ Others push/ drive a wheelchair / cart |
| □ Self-driven wheelchair (self-driven) |
| □ electrically powered wheelchair |
| □ walker aid |
| □ Other _________________* |

Depends on option 6 of question 8

**10. When the child is training in the rehabilitation center, the frequency of stretch training is [single choice] ***

| ○ <5 times / week |
| --- |
| ○ 5 times / week |

Depends on option 2 of question 8

**11 whether the child rehabilitated at home? [single choice] ***

| ○ Almost every day (> 5 days / week) |
| --- |
| ○ Frequent (3-5 days / week) |
| ○ Occasional (1-2 days / week) |
| ○ never |

**12. The reason why you do not have family rehabilitation is [multiple choice] ***

| □ Lack of relevant technical training and knowledge |
| --- |
| □ Lack of environment and conditions(necessary equipment etc.) |
| □ Lack of manpower / time / money |
| □ Other _________________* |

Depends on option 4 in question 11

**13 What rehabilitation treatment has your child conducted at home? [multiple choice] ***

| □ Stretch training |
| --- |
| □ Motor training |
| □ Respiratory care and sputum discharge |
| □ Swallowing function training / oral sensory stimulation |
| □ Position management, posture setting |
| □ Orthotics and auxiliary equipment wearing |
| □ Other _________________* |

Depends on the 1; 2; 3 options in question 11

**14. Which rehabilitation program is most useful / most important in your opinion: [Multiple choice] ***

| □ Physical factor therapy (such as electrical stimulation, magnetic stimulation, etc.) |
| --- |
| □ Stretch training |
| □ Motor training |
| □ Respiratory function training (autonomous cough training, auxiliary sputum discharge training, respiratory muscle training, etc.) |
| □ Swallowing function training / oral sensory stimulation |
| □ Use auxiliary equipment or wear orthotics |
| □ Occupational therapy |
| □ Psychotherapy |
| □ nutritional care |
| □ Traditional Chinese medicine therapy (acupuncture etc.) |
| □ Other _________________* |
| □ All treatment above have not obvious effects |

Depends on option 1 in question 1

**15. The biggest difficulty in the rehabilitation treatment in your opinion is [multiple choice] ***

| □ The economic burden |
| --- |
| □ Difficulty of appointment in the rehabilitation facility / long distance / inconvenient transportation |
| □ Poor efficacy / no visible effect |
| □ Time cost is too much/ lack of time |
| □ Child unwilling to cooperate training |
| □ Can not customize to the appropriate accessories |
| □ Other reasons: _________________* |

Depends on option 1 in question 1

**Part3:** **Patient-reported outcome**

**1. Number of emergency hospitalizations for emergencies in the past year (****excluding planned sheath injection, regular follow-up, spinal orthopedic surgery, gastrostomy, etc.) [Single choice] ***

| ○ 2 times / year |
| --- |
| ○ 3-5 times / year |
| ○> 5 times / year |

**2. Which interval below is the current age of the patient [single choice] ***

| ○ <1 year old |
| --- |
| ○ 1-2 years old |
| ○> 2 years old |

**3. PedsQL NMM (PedsQL™ Neuromuscular Module, age 2-18 years)**The table below lists some problems that may trouble the child. Please indicate the extent to which each question has bothered your child in the past month [Matrix Scale question] *

|  | never | Almost never | Sometimes | Often | Almost always |
| --- | --- | --- | --- | --- | --- |
| 1. It is hard for my child to breathe | ○ | ○ | ○ | ○ | ○ |
| 2. My child gets sick easily | ○ | ○ | ○ | ○ | ○ |
| 3. My child gets sores and/or rashes | ○ | ○ | ○ | ○ | ○ |
| 4. My child’s legs hurt | ○ | ○ | ○ | ○ | ○ |
| 5. My child feels tired | ○ | ○ | ○ | ○ | ○ |
| 6. My child’s back feels stiff | ○ | ○ | ○ | ○ | ○ |
| 7. My child wakes up tired | ○ | ○ | ○ | ○ | ○ |
| 8. My child’s hands are weak | ○ | ○ | ○ | ○ | ○ |
| 9. It is hard for my child to use the bathroom | ○ | ○ | ○ | ○ | ○ |
| 10. . It is hard for my child to gain or lose weight when he or  she wants to | ○ | ○ | ○ | ○ | ○ |
| 11. It is hard for my child to use his or her hands | ○ | ○ | ○ | ○ | ○ |
| 12 It is hard for my child to swallow food | ○ | ○ | ○ | ○ | ○ |
| 13. It takes my child a long time to bathe or shower | ○ | ○ | ○ | ○ | ○ |
| 14. My child gets hurt accidentally | ○ | ○ | ○ | ○ | ○ |
| 1. My child takes a long time to eat | ○ | ○ | ○ | ○ | ○ |
| 16. It is hard for my child to turn him or herself during the  night | ○ | ○ | ○ | ○ | ○ |
| 17.It is hard for my child to go places with his or her  equipment | ○ | ○ | ○ | ○ | ○ |
| 18.It is hard for my child to tell the doctors and nurses  how he or she feels | ○ | ○ | ○ | ○ | ○ |
| 19. It is hard for my child to ask the doctors and nurses  questions | ○ | ○ | ○ | ○ | ○ |
| 20. It is hard for my child to explain his or her illness to  other people | ○ | ○ | ○ | ○ | ○ |
| 21. It is hard for our family to plan activities like vacations | ○ | ○ | ○ | ○ | ○ |
| 22. It is hard for our family to get enough rest | ○ | ○ | ○ | ○ | ○ |
| 23. I think money is a problem in our family | ○ | ○ | ○ | ○ | ○ |
| 1. I think our family has a lot of problems | ○ | ○ | ○ | ○ | ○ |
| 25.My child does not have the equipment he or she needs | ○ | ○ | ○ | ○ | ○ |

Depends on option 3 of question 2

**4. PedsQL Baby Scale (PedsQL Infant Scales, 13-24 months )**The table below lists some problems that may bother the child. Please indicate the extent to which each question has bothered your child in the past month [Matrix Scale question] *

|  | never | Almost never | Sometimes | Often | Almost always |
| --- | --- | --- | --- | --- | --- |
| 1. Low energy level | ○ | ○ | ○ | ○ | ○ |
| 2. Difficulty participating in active play | ○ | ○ | ○ | ○ | ○ |
| 3. Having hurts or aches | ○ | ○ | ○ | ○ | ○ |
| 4. Feeling tired | ○ | ○ | ○ | ○ | ○ |
| 5. Being lethargic | ○ | ○ | ○ | ○ | ○ |
| 6. Resting a lot | ○ | ○ | ○ | ○ | ○ |
| 7. Feeling too tired to play | ○ | ○ | ○ | ○ | ○ |
| 8. Difficulty walking | ○ | ○ | ○ | ○ | ○ |
| 9. Difficulty running a short distance without falling | ○ | ○ | ○ | ○ | ○ |
| 10. Having gas | ○ | ○ | ○ | ○ | ○ |
| 11. Spitting up after eating | ○ | ○ | ○ | ○ | ○ |
| 12. Difficulty breathing | ○ | ○ | ○ | ○ | ○ |
| 13. Being sick to his/her stomach | ○ | ○ | ○ | ○ | ○ |
| 14. Difficulty swallowing | ○ | ○ | ○ | ○ | ○ |
| 15. Being constipated | ○ | ○ | ○ | ○ | ○ |
| 16. Having a rash | ○ | ○ | ○ | ○ | ○ |
| 17. Having diarrhea | ○ | ○ | ○ | ○ | ○ |
| 18. Wheezing | ○ | ○ | ○ | ○ | ○ |
| 19. Vomiting | ○ | ○ | ○ | ○ | ○ |
| 20. Feeling afraid or scared | ○ | ○ | ○ | ○ | ○ |
| 21. Feeling angry | ○ | ○ | ○ | ○ | ○ |
| 22. Crying or fussing when left alone | ○ | ○ | ○ | ○ | ○ |
| 23. Difficulty soothing himself/herself when upset | ○ | ○ | ○ | ○ | ○ |
| 24. Difficulty falling asleep | ○ | ○ | ○ | ○ | ○ |
| 25. Crying or fussing while being cuddled | ○ | ○ | ○ | ○ | ○ |
| 26. Feeling sad | ○ | ○ | ○ | ○ | ○ |
| 27. Difficulty being soothed when picked up or held | ○ | ○ | ○ | ○ | ○ |
| 28. Difficulty sleeping mostly through the night | ○ | ○ | ○ | ○ | ○ |
| 29. Crying a lot | ○ | ○ | ○ | ○ | ○ |
| 30. Feeling cranky | ○ | ○ | ○ | ○ | ○ |
| 31. Difficulty taking naps during the day | ○ | ○ | ○ | ○ | ○ |
| 32. Not smiling at others | ○ | ○ | ○ | ○ | ○ |
| 33. Not laughing when tickled | ○ | ○ | ○ | ○ | ○ |
| 34. Not making eye contact with a caregiver | ○ | ○ | ○ | ○ | ○ |
| 35. Not laughing when cuddled | ○ | ○ | ○ | ○ | ○ |
| 36. Being uncomfortable around other children | ○ | ○ | ○ | ○ | ○ |
| 37. Not imitating caregivers’ actions | ○ | ○ | ○ | ○ | ○ |
| 38. Not imitating caregivers’ facial expressions | ○ | ○ | ○ | ○ | ○ |
| 39. Not imitating caregivers’ sounds | ○ | ○ | ○ | ○ | ○ |
| 40. Not able to fix his/her attention on objects | ○ | ○ | ○ | ○ | ○ |
| 41. Not imitating caregivers’ speech | ○ | ○ | ○ | ○ | ○ |
| 42. Difficulty pointing to his/her body parts when asked | ○ | ○ | ○ | ○ | ○ |
| 43. Difficulty naming familiar objects | ○ | ○ | ○ | ○ | ○ |
| 44. Difficulty repeating words | ○ | ○ | ○ | ○ | ○ |
| 45. Difficulty keeping his/her attention on things | ○ | ○ | ○ | ○ | ○ |

Depends on option 2 of question 2

**5. PedsQL Baby Scale (PedsQL Infant Scales, 1-12months)**The table below lists some problems that may bother the child. Please indicate the extent to which each question has bothered your child in the past month [Matrix Scale question] *

|  | never | Almost never | Sometimes | Often | Almost always |
| --- | --- | --- | --- | --- | --- |
| 1. Low energy level | ○ | ○ | ○ | ○ | ○ |
| 2. Difficulty participating in active play | ○ | ○ | ○ | ○ | ○ |
| 3. Having hurts or aches | ○ | ○ | ○ | ○ | ○ |
| 4. Feeling tired | ○ | ○ | ○ | ○ | ○ |
| 5. Being lethargic | ○ | ○ | ○ | ○ | ○ |
| 6. Resting a lot | ○ | ○ | ○ | ○ | ○ |
| 7. Having gas | ○ | ○ | ○ | ○ | ○ |
| 8. Spitting up after eating | ○ | ○ | ○ | ○ | ○ |
| 9. Difficulty breathing | ○ | ○ | ○ | ○ | ○ |
| 10. Being sick to his/her stomach | ○ | ○ | ○ | ○ | ○ |
| 11. Difficulty swallowing | ○ | ○ | ○ | ○ | ○ |
| 12. Being constipated | ○ | ○ | ○ | ○ | ○ |
| 13. Having a rash | ○ | ○ | ○ | ○ | ○ |
| 14. Having diarrhea | ○ | ○ | ○ | ○ | ○ |
| 15. Wheezing | ○ | ○ | ○ | ○ | ○ |
| 16. Vomiting | ○ | ○ | ○ | ○ | ○ |
| 17. Feeling afraid or scared | ○ | ○ | ○ | ○ | ○ |
| 18. Feeling angry | ○ | ○ | ○ | ○ | ○ |
| 19. Crying or fussing when left alone | ○ | ○ | ○ | ○ | ○ |
| 20. Difficulty soothing himself/herself when upset | ○ | ○ | ○ | ○ | ○ |
| 21. Difficulty falling asleep | ○ | ○ | ○ | ○ | ○ |
| 22. Crying or fussing while being cuddled | ○ | ○ | ○ | ○ | ○ |
| 23. Feeling sad | ○ | ○ | ○ | ○ | ○ |
| 24. Difficulty being soothed when picked up or held | ○ | ○ | ○ | ○ | ○ |
| 25. Difficulty sleeping mostly through the night | ○ | ○ | ○ | ○ | ○ |
| 26. Crying a lot | ○ | ○ | ○ | ○ | ○ |
| 27. Feeling cranky | ○ | ○ | ○ | ○ | ○ |
| 28. Difficulty taking naps during the day | ○ | ○ | ○ | ○ | ○ |
| 29. Not smiling at others | ○ | ○ | ○ | ○ | ○ |
| 30. Not laughing when tickled | ○ | ○ | ○ | ○ | ○ |
| 31. Not making eye contact with a caregiver | ○ | ○ | ○ | ○ | ○ |
| 32. Not laughing when cuddled | ○ | ○ | ○ | ○ | ○ |
| 33. Not imitating caregivers’ actions | ○ | ○ | ○ | ○ | ○ |
| 34. Not imitating caregivers’ facial expressions | ○ | ○ | ○ | ○ | ○ |
| 35. Not imitating caregivers’ sounds | ○ | ○ | ○ | ○ | ○ |
| 36. Not able to fix his/her attention on objects | ○ | ○ | ○ | ○ | ○ |

Depends on option 1 of question 2
