# Supplementary figures and images for: Rehabilitation for spinal muscular atrophy patients in China: a national cross-sectional study
Source: Orphanet J Rare Dis. 2024 Jul 25;19:279. doi: 10.1186/s13023-024-03291-x (PMC11282710; doi:10.1186/s13023-024-03291-x)

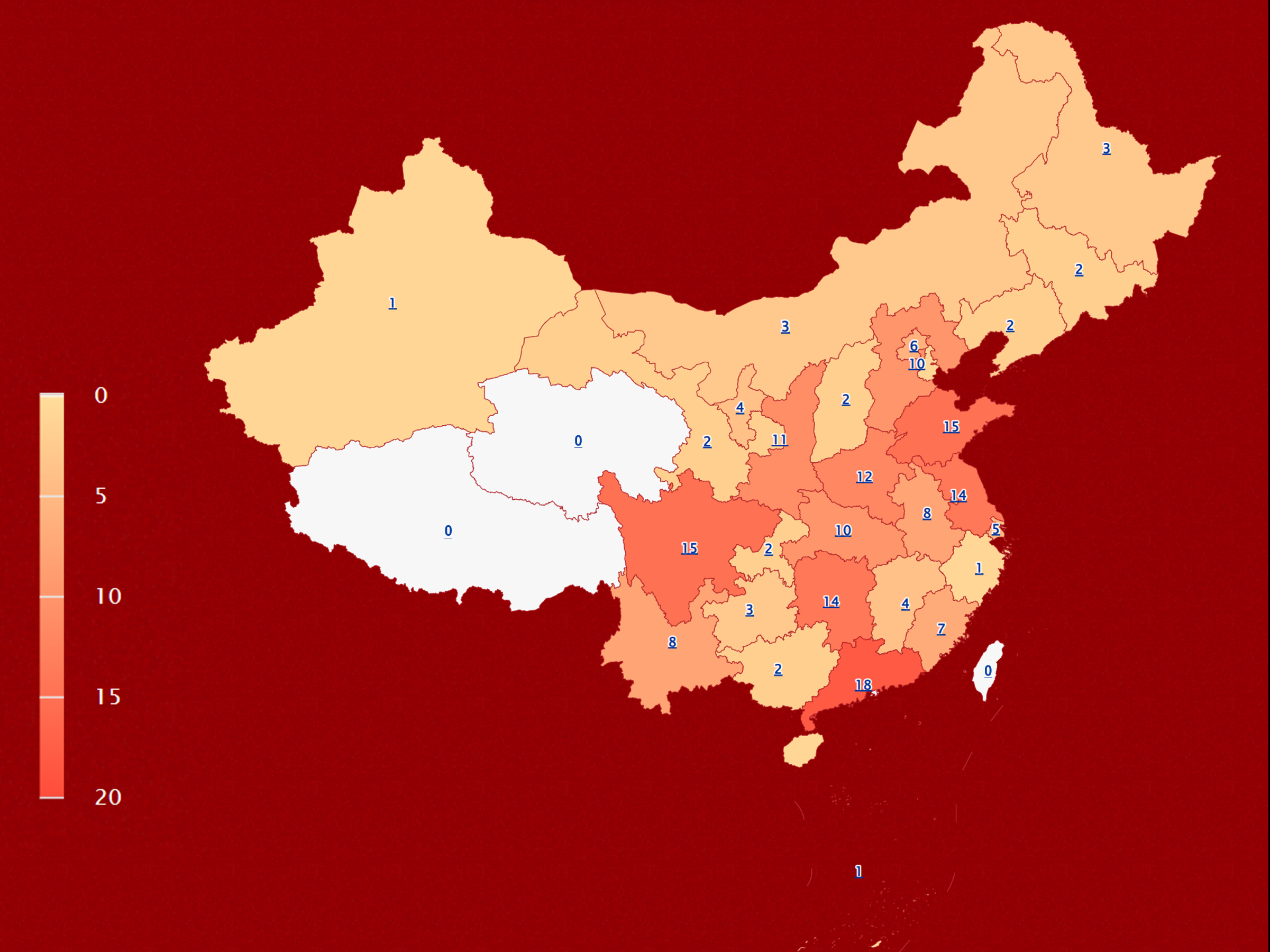

Supplement: Supplementary file 2 — Supplementary Material 2: The national distribution of patients [file 13023_2024_3291_MOESM2_ESM.jpg]
